# Development and validation of a predictive model for colorectal polyps in patients with NAFLD based on risk factors identified from a meta-analysis

**DOI:** 10.3389/fmed.2026.1838023

**Published:** 2026-07-21

**Authors:** Dong Zhai, Miaofang Feng, Xiaojuan Tong

**Affiliations:** 1 The Third Affiliated Hospital of Zhejiang Chinese Medical University (Zhongshan Hospital of Zhejiang Province), Hangzhou, China; 2 Shangyu Traditional Chinese Medicine Hospital, Shaoxing, China; 3 The First Affiliated Hospital of Zhejiang Chinese Medical University (Zhejiang Provincial Hospital of Chinese Medicine), Hangzhou, China

**Keywords:** colorectal polyps, development, meta-analysis, non-alcoholic fatty liver disease, prediction model, validation

## Abstract

**Objective:**

Colorectal polyps are precancerous lesions of colorectal cancer. The incidence of colorectal polyps in patients with non-alcoholic fatty liver disease (NAFLD) is significantly higher than that in normal people, but the underlying risk factors for its occurrence are not yet completely clear. The purpose of this study is to establish and validate a risk prediction model for colorectal polyps in NAFLD, in order to conduct early identification and intervention for high-risk populations.

**Methods:**

We conducted a meta-analysis to identify the eligible studies that met the inclusion and exclusion criteria, and extracted the potential candidate risk factors for colorectal polyps in patients with NAFLD. Subsequently, a retrospective collection was conducted of patients with NAFLD who underwent colonoscopy at Zhejiang Provincial Hospital from 2023 to 2025. These patients were divided into a training set and a validation set in a 7:3 ratio. Based on the candidate risk factors identified from the meta-analysis, predictive factors were determined using LASSO regression and multivariable logistic regression in the training set, and a nomogram was subsequently constructed. The performance of the model was evaluated using the area under the receiver operating characteristic curve (AUC), calibration curve analysis, and decision curve analysis (DCA).

**Results:**

The meta-analysis for identifying candidate risk factors included 7,200 patients with NAFLD, of whom 3,686 had colorectal polyps. Through the combined effect size, subgroup and sensitive analysis, 8 risk factors were selected as candidate risk factors. After LASSO regression and multivariable logistic regression in the training set, six predictors were finally determined for model construction: age ≥ 50, male sex, obesity, diabetes, hypertriglyceridemia, and fatty liver type. Our nomogram model demonstrated acceptable calibration and discrimination in both training and validation sets (AUCs: 0.73 and 0.71). The DCA curve indicated that the nomogram provided a potential net benefit in predicting colorectal polyps in patients with NAFLD.

**Conclusion:**

This predictive model can identify NAFLD patients at risk of colorectal polyps, aiding early intervention and improving prognosis.

## 1 Introduction

Colorectal cancer is a common malignancy. According to the latest global cancer statistics, its incidence now ranks as the third highest, and its mortality rate as the second highest (1, 2). The development of colorectal cancer follows the “adenoma-carcinoma” sequence, in which precancerous lesions (primarily adenomatous polyps) progress to colorectal cancer over a period of 10–15 years (3). NAFLD has emerged as the most common type of chronic liver disease worldwide (4). Patients with NAFLD have an increased risk for colorectal polyps and adenomas compared with the general population (5, 6). Early detection of colorectal polyps (especially adenomas) in patients with NAFLD is of vital importance for preventing colorectal cancer. (7). Colonoscopy is the gold standard for screening colorectal cancer, adenomas, and polyps. However, the burden of bowel preparation often leads to poor patient adherence, causing precancerous lesions to be missed and ultimately progress to malignancy (8). Therefore, identifying the modifiable risk factors for colorectal cancer and colon polyps is of great significance for diagnosis and prevention of disease progression.

While numerous studies have investigated potential risk factors, such as age, gender, smoking, insulin resistance, and metabolic syndrome, for colorectal polyps in patients with NAFLD (9, 10), the results remain heterogeneous, largely attributable to variations in sample size and diagnostic criteria.

The nomogram is a reliable predictive model that uses an intuitive graphical format to quantify clinical event risks (11). This study first identified the risk factors through a meta-analysis, and then used independent hospital data to construct and validate the predictive model. The ultimate goal is to facilitate risk stratification and personalized screening strategies, aiming to mitigate the burden of colorectal cancer in this specific population.

## 2 Materials and methods

### 2.1 Study populations

#### 2.1.1 Derivation cohort

The derivation cohort were obtained through a meta-analysis and systematic review. Potential risk factors for colorectal polyps in patients with NAFLD were identified by systematically searching three major databases: Pubmed, Embase, and the Cochrane Library, up to and including April 1, 2025. Search key keywords included: “Non-alcoholic Fatty Liver Disease,” “Intestinal Polyps,” “Polyps,” “Colonic Polyps,” “Adenomatous Polyps.” Detailed search strategies were provided in the supplementary methods section.

**FIGURE 1:**
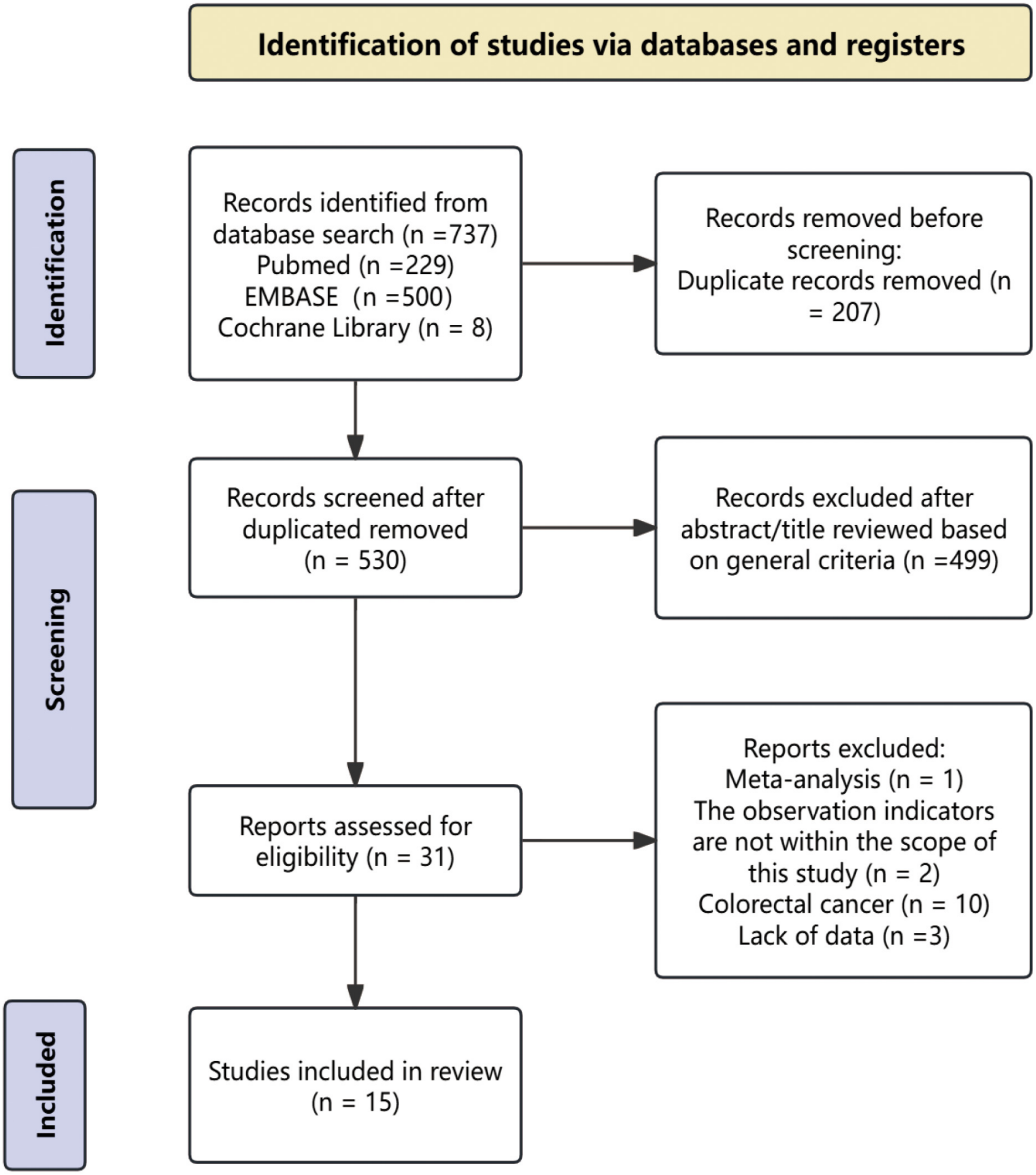
PRISMA Flow chart for meta-analysis.

The inclusion criteria of this study are as follows: (1) Adult patients (over 18 years old) diagnosed with NAFLD/NASH through Liver ultrasound, abdominal CT and liver biopsy, (2) cohort, case-control and cross-sectional studies, (3) Studies that have at least one factor related to colorectal polyps; (4) The results should include the odds ratio (OR), risk (RR), and 95% confidence interval (CI) of the risk ratio (HR), or sufficient data to calculate them, (5) Published in English. Studies with repeated information, animal experiments, meta-analyses, reviews, conference proceedings, case reports and unavailable data were excluded. The meta-analysis was registered in PROSPERO (CRD420251040937). XJ Tong and D Zhai independently conducted literature retrieval and data extraction. Any differences are resolved by reaching a consensus through discussion. We evaluated the quality and risk of bias of included studies using the Newcastle-Ottawa Scale (NOS), classifying studies scoring ≥ 7 as high-quality. Literature Screening Process were displayed in Figure 1.

#### 2.1.2 Validation cohort

Patients with NAFLD who underwent colonoscopy at The First Affiliated Hospital of Zhejiang Chinese Medical University from January 2023 to December 2025. Inclusion criteria included: (1) age over 18 years, (2) Liver B-ultrasound or CT scan or liver biopsy indicates NAFLD or NASH, (3) Complete colonoscopy examination. Exclusion criteria included: (i) Patients without complete case information and clinical data. (ii) Colorectal cancer or previous colorectal cancer surgery. (iii) chronic viral hepatitis B, cirrhosis, and liver cancer. (iv) Other tumors or organ failure (Cardiac function grade III or IV, or Child-Pugh grade C, renal dialysis). To be consistent with the meta-analysis and WHO Asia-Pacific guidelines, obesity was defined as BMI ≥ 24 (12, 13). This threshold has been validated as optimal for identifying metabolic risk factors (14). NAFLD was diagnosed by ultrasound based on the following criteria: (1) liver echogenicity greater than that of the renal cortex; (2) near-field echo dense enhancement with far-field attenuation; (3) indistinct intrahepatic duct structures, especially the hepatic veins; and (4) mild to moderate hepatomegaly. NASH is diagnosed through liver biopsy. Missing data were addressed through multiple imputation.

This study has received approval from the Ethics Committee of the First Affiliated Hospital of Zhejiang Chinese Medicine University (Zhejiang Hospital of Traditional Chinese Medicine), with the reference number 2025-KLS-39s-02 and 2026-KLS-339-02. This study is registered at http://60.191.20.70:8888/IRB/ (MR-50-24-042059/MR-33-26-054501).

### 2.2 Statistical analysis

#### 2.2.1 Factors selection from Meta-analysis

Review Manager 5.3 software was utilized to perform Meta-analysis. The heterogeneity results of each study were evaluated using the *p*-values from the I^2^ and Cochrane Q tests. If I^2^ is greater than 50% or the *p*-value of Cochrane Q test is less than 0.05, it indicates that there is significant heterogeneity among the samples. In this case, the random effects model should be used; otherwise, the fixed effects model should be employed.

To obtain meaningful risk factors, we first extracted and summarized the OR and their 95% CI of all risk factors based on the results selected from meta-analysis. Factors exhibiting significant heterogeneity (*I*^2^> 50%) underwent subgroup analysis. The stability of meta-analysis results was evaluated via sensitivity analyses using sequential study removal. Selection criteria for candidate factors: factors determined through meta-analysis, subgroup analysis, sensitivity analysis (*P* < 0.05), factors consistently supported in the literature review, and factors with clinical practicality.

#### 2.2.2 Predictors identification, model development and validation

The retrospective cohort was randomly split into a training set (70%) and a validation set (30%). The training set was used for model development, and the validation set for internal validation. Candidate risk factors identified from the meta-analysis were screened using LASSO regression were first screened using LASSO regression in the training set. The optimal λ value was determined by 10-fold cross-validation. Furthermore, a multivariable stepwise logistic regression was utilized to construct the final prediction model for colorectal polyps in patients with NAFLD, using the potential predictors identified through LASSO regression.

We constructed a nomogram using the statistically significant predictors obtained from the stepwise logistic regression through the R software (version 4.3.2). Model discrimination was assessed using the area under the receiver operating characteristic curve (AUC), with values ranging from 0.5 (no discrimination) to 1 (perfect discrimination). Clinical utility was evaluated using decision curve analysis (DCA), and model calibration was examined using the Hosmer-Lemeshow goodness-of-fit test.

## 3 Results

### 3.1 Description of the cohorts

#### 3.1.1 Derivation cohort

A total of 7,200 NAFLD patients enrolled in the study by meta-analysis, published between 2009 and 2024, with colorectal polyps detected in 3,686 (51.19%). Regarding the geographical distribution of the 15 included studies, 11 were from Asia (China, South Korea, and Israel), 3 from the Americas (the USA and Peru), and 1 from Oceania (Australia), as shown in Table 1. A total of 12 risk factors were identified (including age, gender, smoking history, and diabetes, etc.), with detailed information summarized in Tables 1, 2. The quality of the 15 studies was all good because the scores in the Newcastle-Ottawa assessment criteria were all above 7 points (Table 1).

**TABLE 1:** The basic characteristics of the 15 studies and the evaluation results of the literature quality.

References	Country	Age (year)	Sex (male/ female)	Study type	Polyps in NAFLD/NASH	Adjusted confounding factors	NOS
Yang et al. (6)	China	54.4 ± 14.0	1,728/1,300	Retrospective cross-sectional	926	Age, sex, TG, TC, LDL-C, HDL-C	9
Mahamid et al. (18)	Israel	41.1 ± 12.5	115/108	Retrospective cohort observational	14	Gender, age, C-reactive protein, smoking	8
Hwang et al. (19)	Korea	47.0 ± 9.0	1,911/2,917	Cross-sectional	298	Age, gender, smoking, NAFLD, metabolic syndrome, hypertension and diabetes	9
Cho et al. (20)	California	55.9 ± 12.9	154/169	Cohort	133	Age, sex, Diabete mellitus, Hypertension, NAFL, NASH	9
Chen et al. (21)	China	47.5 ± 10.6	2,430/1,256	Retrospective cross-sectional	492	Gender, age, smoking, alcohol, MS.	9
Chao et al. (22)	China	49.4 ± 8.2	469/249	Retrospective	81	Gender, age, BMI, WC, FBG, SUA, TG, Fatty liver disease	9
Blackett et al. (23)	USA	60.4 ± 8.9	183/186	Retrospective cross-sectional	90	Rates of hyper-lipidemia, diabetes, obesity	9
Bhatt et al. (24)	USA	59.8	398/193	Retrospective cohort	40	Age, NAFLD, Alcohol use	9
Seo et al. (13)	Korea	52.4 ± 9.1	2,130/1,311	Retrospective cohort	764	Age, sex, smoking, triglyceride level, HDL cholesterol level, hypertension, visceral fat area, diabetes, and body mass index	7
Li et al. (5)	China	54.5 ± 0.6	566/523	Retrospective cohort	142	Sex, NAFLD, CAP, body mass index, triglyceride, aspartate aminotransferase, fasting plasma glucose	9
Huang et al. (25)	China	53.7 ± 9.	890/612	Retrospective cohort	120	Age, Body mass index, Male gender, NAFLD, Smoking, Hypertension, Diabetes mellitus	9
BardalesZuta et al. (26)	Peru	69	69/69	Case-control	45	Sex, Age, Hypertension, Diabetes, Dyslipidemia, Obesity, NAFLD	9
Stadlmayr et al. (27)	Austria	60.88 ± 9.98	603/608	Cohort	215	Sex, age, body mass index, Liver steatosis, Glucose intolerance	8
Fliss-Isakov et al. (28)	Israel	58.4 ± 6 6.6	416/412	Case-control	162	Obesity, abdominal obesity, Hypertension, IFG, High HbA1c%, Hypertriglyceridemia, Low HDL cholesterol, High TG/HDL ratio, NAFLD, Metabolic syndrome	8
	China	57	51,328/33,122	Case-control	161	Sex, age, NAFLD, Chronic kidney disease	7

**TABLE 2:** The risk factors identified through meta-analysis.

Factors	Study(n)	Pooled result		*I* ^2^
		OR (95%CI)	*P*	
Age	10	1.04 (1.03–1.06)	0.000	91.7%
Sex	11	1.91 (1.37–2.67)	0.000	93.2%
Smoking	7	1.39 (1.19–1.63)	0.000	70.3%
Hypertension	6	1.30 (1.06–1.58)	0.011	53.8%
Diabetes	6	1.27 (1.10–1.46)	0.001	0.0%
BMI	4	1.07 (0.99–1.14)	0.074	76.5%
MS	4	1.24 (1.01–1.51)	0.04	56%
Raised FBS	1	1.24 (1.00–1.54)	0.292	58.4%
Hypertriglyceridemia	2	1.24 (1.04–1.49)	0.002	0.0%
Low HDL	3	1.03 (0.75–1.42)	0.34	0.0%
Uric acid	2	1.00 (1.00–1.00)	0.407	60.9%
Fatty liver type	14	1.49 (1.39–1.60)	0.000	78.1%

#### 3.1.2 validation cohort

We conducted a retrospective analysis of 609 patients with NAFLD who underwent colonoscopy. Among these patients, 460 were found to have colorectal polyps, representing 75.53% of the cohort. The mean age of all participants was 55.89 ± 10.80 years, and 375 (61.58%) were male. The patients were categorized into two groups according to the presence or absence of colorectal polyps: 460 with both NAFLD and colorectal polyps, and 149 with NAFLD but without polyps. Compared to the group without polyps, the group with polyps showed higher proportions of the following factors: smoking history (25.43% vs. 20.13%), hypertension (43.48% vs. 32.21%), diabetes (25.87% vs. 12.08%), obesity (71.96% vs. 70.47%), hypertriglyceridemia (73.70% vs. 53.69%), low HDL (46.52% vs. 37.58%), and NASH (20.22% vs. 2.01%). The baseline characteristics of the patients in the verification queue are detailed in Table 3.

**TABLE 3:** Characteristics of validation cohort.

Variable n (%)	Total (*n* = 609)	NAFLD with polyp group (*n* = 460)	NAFLD without polyp group (*n* = 149)	Statistic (χ ^2^)	*P*
Age, n (%)				7.49	0.00
≥ 50	445 (73.07)	349 (75.87)	96 (64.43)		
<50	164 (26.93)	111 (24.13)	53 (35.57)		
Sex, n (%)				7.10	0.08
Male	375 (61.58)	297 (64.60)	78 (52.35)		
Female, n (%)	234 (31.42)	163 (35.40)	71 (47.65)		
Smoking, n (%)				1.73	0.19
No	462 (75.86)	343 (74.57)	119 (79.87)		
Yes	147 (24.14)	117 (25.43)	30 (20.13)		
Hypertension, n (%)				5.92	0.02
No	361 (59.28)	260 (56.52)	101 (67.79)		
Yes	248 (40.72)	200 (43.48)	48 (32.21)		
Diabetes mellitus, n (%)				12.27	0.00
No	472 (77.50)	341 (74.13)	131 (87.92)		
Yes	137 (22.50)	119 (25.87)	18 (12.08)		
Obesity, n (%)				0.12	0.73
No	173 (28.41)	129 (28.04)	44 (29.53)		
Yes	436 (71.59)	331 (71.96)	105 (70.47)		
Hypertriglyceridemia,n (%)				0.32	0.57
No	245 (40.23)	121 (26.30)	69 (46.31)		
Yes	364 (59.77)	339 (73.70)	80 (53.69)		
Low HDL, n (%)				3.64	0.06
No	339 (55.67)	246 (53.48)	93 (62.42)		
Yes	270 (44.33)	214 (46.52)	56 (37.58)		
Fatty liver type, n (%)				28.09	0.00
NAFLD	513 (84.73)	367 (79.78)	146 (97.99)		
NASH	96(15.27)	93 (20.22)	3 (2.01)		

### 3.2 Development and validation of the predictive model

**FIGURE 2:**
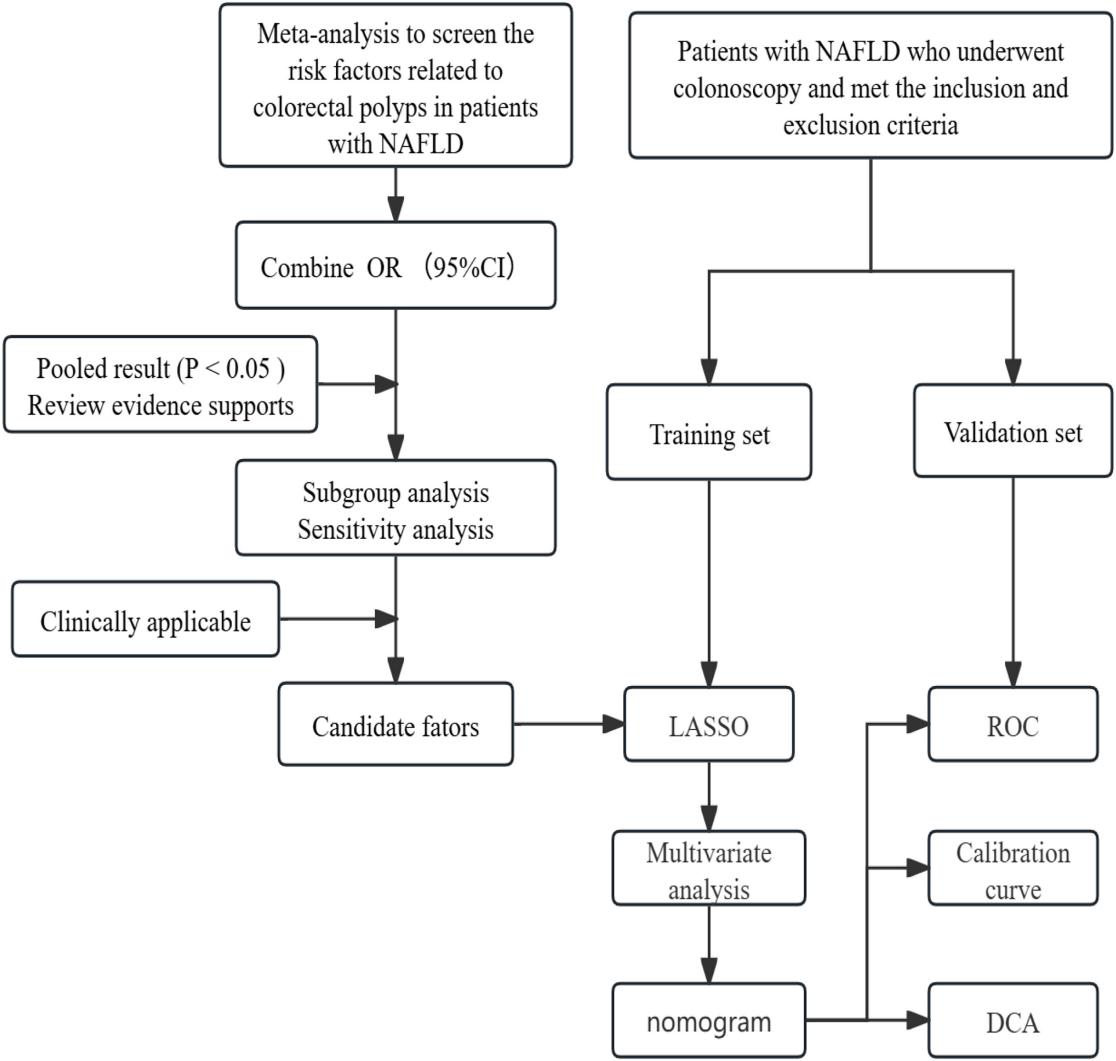
Study flow diagram.

The process of predictor factors selection and model construction is shown in Figure 2. According to systematic reviews and meta-analyses, 8 of the 12 factors with *p* < 0.05: age, sex, smoking, hypertension, diabetes, Metabolic syndrome, hypertriglyceridemia, fatty liver type, as shown in Table 1. Metabolic syndrome, along with obesity, hypertriglyceridemia, and low HDL, all indicate metabolic disorders. Therefore, metabolic syndrome was not included in the final model. Furthermore, numerous studies have demonstrated that overweight and obesity are closely related to NAFLD and colorectal polyps (15, 16). Accordingly, we incorporated BMI and conducted subgroup and sensitivity analyses. Finally, 8 factors were defined as initial candidate factors: age, sex, smoking, hypertension, diabetes, BMI, hypertriglyceridemia and type of fatty liver.

Subgroup and sensitivity analyses (Table 4) identified eight initial candidate predictors: age ≥ 50 (OR 2.02, 95% CI 1.92–2.13, *P* < 0.001), male sex (OR 1.72, 1.50–1.96, *P* < 0.01), smoking (OR 1.45, 1.31–1.59, *P* < 0.001), hypertension (OR 1.22, 1.09–1.37, *P* < 0.05), diabetes (OR 1.31, 1.08–1.60, *P* < 0.001), obesity (OR 1.20, 1.01–1.43, *P* < 0.05), hypertriglyceridemia (OR 1.24, 1.04–1.49, *P* < 0.05), and fatty liver type (OR 1.49, 1.39–1.60, *P* < 0.001), as shown in Tables 2, 4. These eight candidate factors entered LASSO regression.

**TABLE 4:** Subgroup and Sensitivity analysis of risk factors.

Subgroup	Category	Pooled result		*I* ^2^
		OR (95%CI)	*P*	
Age				
	≥ 50	2.02 (1.92–2.13)	<0.001	19%
	Sensitive analysis	2.02 (1.98–2.06)	<0.001	0%
Sex				
	Male	1.72 (1.50–1.96)	< 0.01	3%
	Female	1.05 (0.61–1.82)	0.86	84%
	Sensitive analysis	1.45 (1.31–1.59)	<0.001	79%
Smoking				
	Sensitive analysis	1.22 (1.09–1.37)	<0.001	0
Hypertension				
	Sensitive analysis	1.17 (1.02–1.34)	0.02	0
Diabetes				
	Sensitive analysis	1.31 (1.08–1.60)	0.007	0
BMI				
	Obesity	1.20 (1.01–1.43)	0.04	43%
	Sensitive analysis	1.38 (1.04–1.84)	0.02	46%
Hypertriglyceridemia				
	Sensitive analysis	1.24 (1.04–1.49)	0.02	0
Fatty liver type				
	NAFLD	1.46 (1.35–1.58)	*p*<0.001	84%
	NASH	1.74 (1.42–2.14)	*P* < 0.001	0%
	Sensitive analysis	1.45 (1.31–1.62)	0.03	45%

**FIGURE 3:**
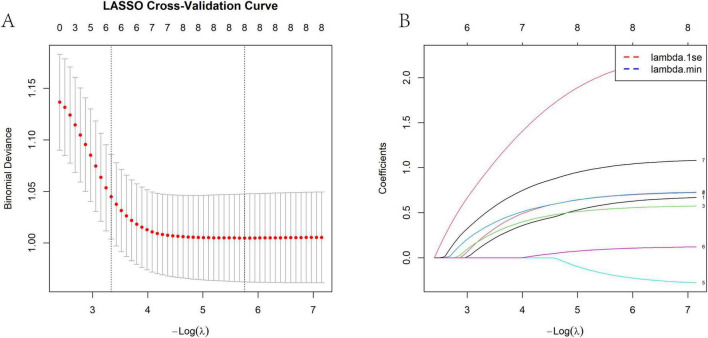
LASSO regression analysis result chart. **(A)** LASSO regression cross validation plot. **(B)** LASSO regression coefficient path diagram.

LASSO regression identified six predictive indicators, which yielded the best model (Figure 3). Multivariate logistic regression (Table 5) confirmed these six factors as independent risk factors for colorectal polyps in NAFLD patients: age ≥ 50 (OR, 2.02; 95% CI, 1.20–3.38), male sex (OR, 1.77; 95%CI, 1.09–2.87), obesity (OR, 1.79; 95%CI, 1.11–2.90), diabetes (OR, 2.71; 95%CI, 1.31–5.62), hypertriglyceridemia (OR, 2.08; 95%CI, 1.29–3.37), and NASH (OR, 4.95; 95% CI, 1.48–16.51), all with *p* < 0.05.

**TABLE 5:** Multivariable regression analysis for colorectal polyps in patients with NAFLD.

Variables	β	S.E	Z	*P*	OR (95%CI)
Intercept	−0.87	0.35	−2.47	0.013	0.42 (0.21 ∼ 0.83)
Age					
< 50					1.00 (Reference)
≥ 50	0.70	0.26	2.66	0.008	2.02 (1.20 ∼ 3.38)
Male					
No					1.00 (Reference)
Yes	0.57	0.25	2.32	0.020	1.77 (1.09 ∼ 2.87)
Obesity					
No					1.00 (Reference)
Yes	0.58	0.25	2.37	0.018	1.79 (1.11 ∼ 2.90)
Diabetes					
No					1.00 (Reference)
Yes	1.00	0.37	2.68	0.007	2.71 (1.31 ∼ 5.62)
Hypertriglyceridemia					
No					1.00 (Reference)
Yes	0.73	0.25	2.98	0.003	2.08 (1.29 ∼ 3.37)
Fitty liver type					
NAFLD					1.00 (Reference)
NASH	1.60	0.62	2.60	0.009	4.95 (1.48 ∼ 16.51)

OR, odds ratio; CI, confidence interval.

**FIGURE 4:**
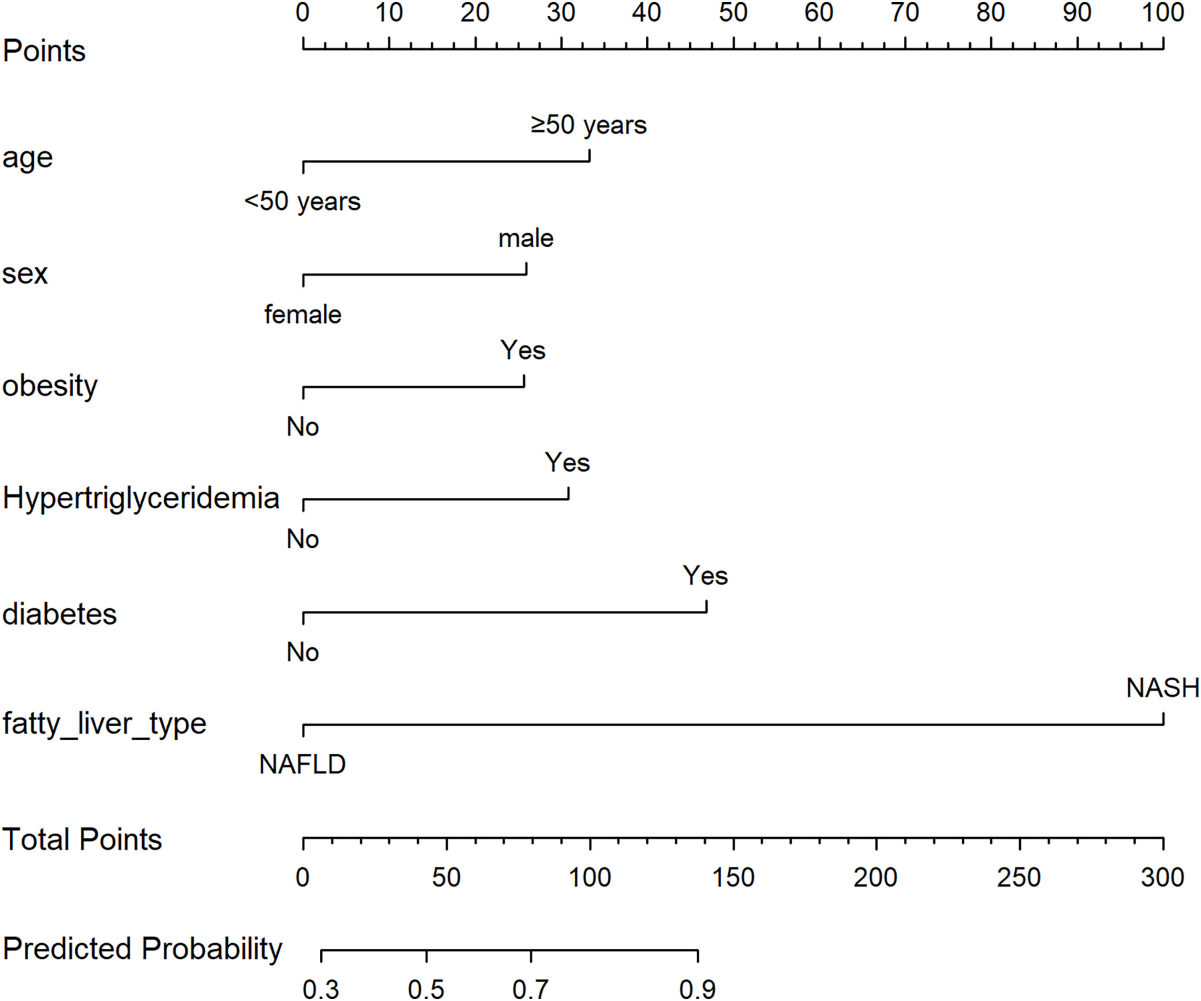
Nomogram for predicting colorectal polyps in patients with nonalcoholic fatty liver disease. Each variable is assigned an individual score. These scores are summed, and the total is then projected onto the risk scale via a vertical line to determine the corresponding risk. Example: A 55-year-old male with obesity, hypertriglyceridemia, diabetes and NASH has a total point of 290, corresponding to a predicted probability of approximately 0.65.

A nomogram incorporating six independent predictors from LASSO regression and multivariable logistic regression was developed for individualized risk assessment of colorectal polyps in NAFLD patients, as shown in Figure 4. Predictors entered into the model were: Age (< 50 vs. ≥ 50), sex (male vs. female), Obesity (yes vs. no), diabetes (yes vs. no), hypertriglyceridemia (yes vs. no), Fatty liver type (NAFLD vs. NASH). This nomogram visualizes the prediction of colorectal polyps in patients with NAFLD. The total points range from 0 to 350. For each predictor, a vertical line is drawn to the point axis, and the corresponding points are recorded. The points from all predictors are then summed. The total points correspond to an overall predicted probability of colorectal polyps, which can be read from the bottom of the nomogram.

**FIGURE 5:**
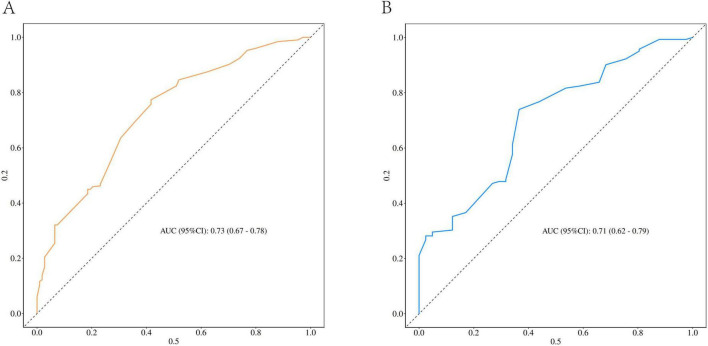
ROC curve for the colorectal polyp risk prediction model in NAFLD patients within the derivation cohorts **(A)** and the validation cohorts **(B)**.

**FIGURE 6:**
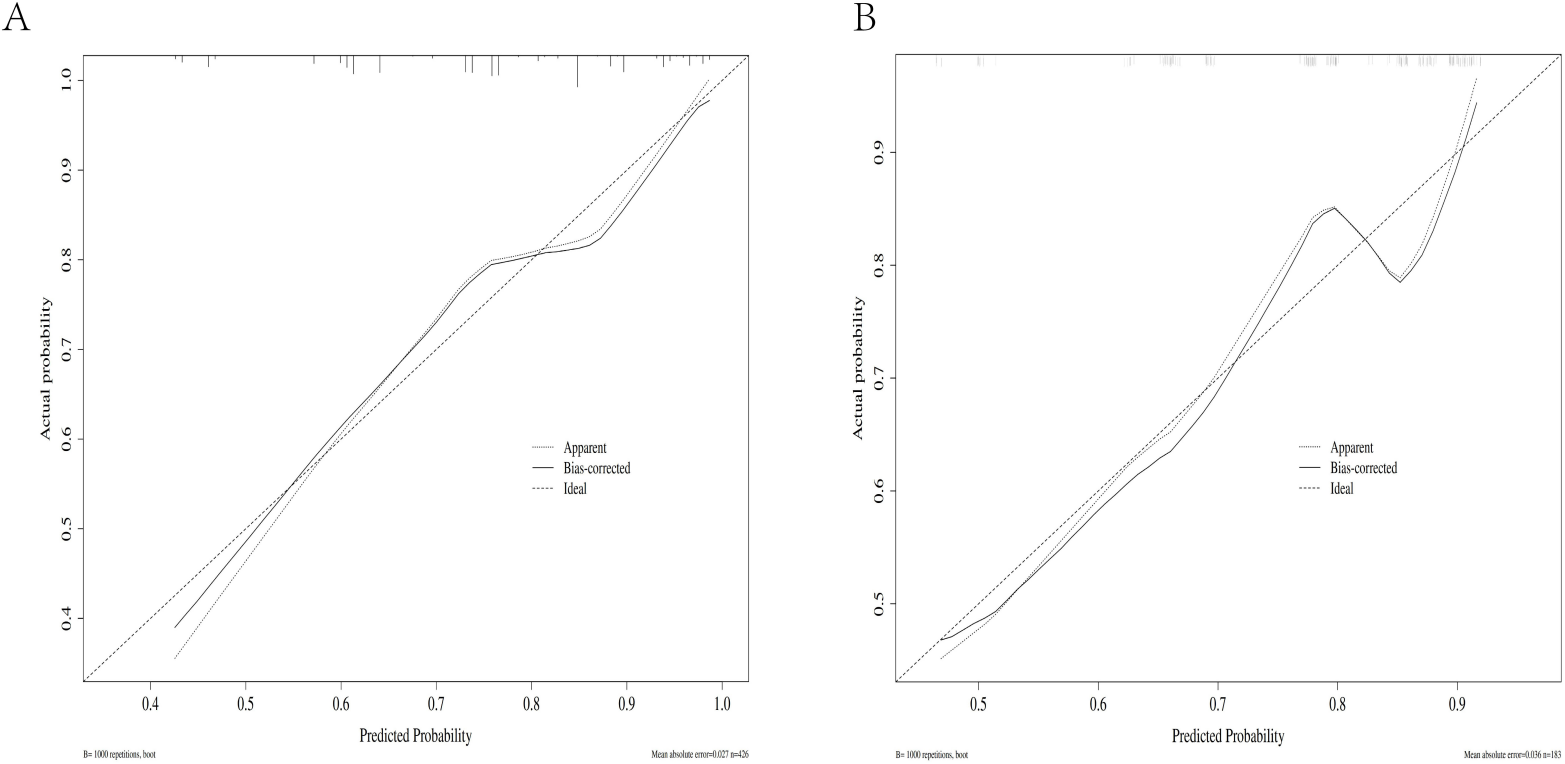
Calibration curves for the nomogram model in the training groups **(A)** and the validation groups **(B)**.

**FIGURE 7:**
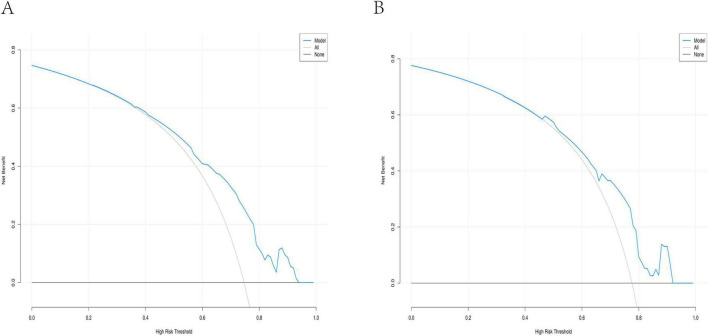
Decision curve evaluation of the nomogram model in the training cohorts **(A)** and validation cohorts **(B)**.

For the training cohort, the AUC was 0.73 (95% CI: 0.67–0.78; Figure 5A), whereas in the validation cohort it was 0.71 (95% CI: 0.62–0.79; Figure 5B), indicating moderate discriminative ability. Bootstrap internal validation with 500 resamples yielded an optimism-corrected AUC of 0.72 for the training set (original AUC 0.73), confirming minimal overfitting. In contrast, an age-only model (Supplementary Figure 1) achieved AUCs of only 0.57 (training set) and 0.54 (validation set), respectively. Calibration curve analysis showed that the predicted probabilities of colorectal polyps in patients with NAFLD were generally consistent with the observed incidences in both the training and validation cohorts (Figures 6A,B). Calibration was acceptable (training intercept 0.00, slope 1.00; validation intercept 0.32, slope 0.76). The optimal probability threshold was determined as 0.653 by maximizing the Youden index, yielding a Youden index of 0.357 (sensitivity 0.774, specificity 0.583) in the training set and 0.329 (sensitivity 0.768, specificity 0.561) in the validation set. The study assessed the model through decision curve analysis (DCA), which demonstrated potential clinical utility in both the derivation (Figure 7A) and validation (Figure 7B) cohorts and showed that the nomogram provided superior net benefit to the age-only model (Supplementary Figure 1).

## 4 Discussion

Colonoscopy is widely regarded as the gold standard for screening colorectal cancer, colorectal polyps, adenomas and associated diseases. However, the high cost and complex bowel preparation required for the procedure pose significant barriers that often deter high-risk patients, leading to missed opportunities for timely therapeutic intervention. NAFLD shares common metabolic risk factors with colorectal polyps and adenomas, including obesity, insulin resistance, diabetes, hyperlipidemia and so on. As a result, patients with NAFLD are at a heightened risk for colorectal polyps even colorectal cancer. Studies have shown that the incidence of colorectal polyps, including adenomas, in NAFLD patients is 1.5 times higher than that in the general population (5, 17). This strong association highlights the importance of identifying specific risk factors within the NAFLD population. Developing a robust predictive model for colorectal polyps would enable accurate identification of high-risk patients, facilitate more targeted colonoscopy screening, and ultimately contribute to the effective prevention of colorectal tumors. Previous research has validated the use of scoring systems as simple and practical tools for the early assessment of colorectal polyps (9). However, no specific assessment model has been developed for colorectal polyps in the NAFLD population. Existing scoring systems, which require CT, MRI, or even liver biopsy, are not feasible for early screening in primary healthcare settings.

Current cohort studies on risk factors for colorectal polyps in patients with NAFLD vary considerably in both sample size and findings. Based on a synthesis of 15 high-quality cohort studies comprising 7,200 patients with NAFLD (5, 6, 13, 18–29), meta-analysis identified eight independent risk factors: age, sex, smoking, hypertension, diabetes, Obesity, Hyper Triglyceride and fatty liver type. After LASSO regression and multivariable logistic regression in the training set, six predictors were finally determined for model construction. The internal validation results demonstrated that the model possesses moderate clinical utility for identifying high-risk NAFLD patients who may benefit from colonoscopy screening.

Advancing age is a well-established risk factor for colorectal polyps. Previous studies have shown that the detection rate among people over 50 years old is 34% higher than that among younger people (30), which corroborates the findings of our study. In older patients with NAFLD, weakened immune function and reduced intestinal mucosal repair capacity may contribute to an increased susceptibility to colorectal polyps and tumors. Therefore, this population warrants close clinical monitoring. Previous studies have demonstrated that male is a risk factor for colorectal polyps. In line with these findings, our study revealed that the incidence of colorectal polyps in male NAFLD patients was 1.9 times higher than in their female counterparts. This disparity likely reflects the greater exposure of men to behavioral risk factors, including smoking, alcohol use, and irregular sleep patterns. Smoking has been proven to be an independent risk factor for colorectal polyps and tumors. In our study, patients with NAFLD who smoked had a 39% higher risk of developing colorectal polyps.

It is noteworthy that diabetes, obesity, hypertriglyceridemia and fatty liver type represent key components of metabolic syndrome. Collectively, their significant association with colorectal polyps highlights the close link between these lesions and metabolic disorders. Strict control of blood sugar and blood lipids has clinical benefits for patients with non-alcoholic fatty liver disease, as high blood lipids and high blood sugar are key factors in the formation of colon polyps. Diabetic patients face a 1.83-fold higher risk (31, 32). In our study, the incidence of colorectal polyps was significantly higher in NAFLD patients with diabetes (OR 2.96). Obesity is also primary predictors in the nomogram. The relationship between obesity and colorectal polyps has been widely discussed. Consistent with the findings of Sato Yumi et al who reported a significantly higher polyp detection rate in overweight individuals (BMI ≥ 24) based on a cohort study of 15,380 participants (33). We also found that elevated BMI was associated with an increased risk of colorectal polyps in NAFLD cohort (OR 1.81). Hyperlipidemia, particularly hypertriglyceridemia plays a key role in the development and progression of colorectal polyps. In our study, hypertriglyceridemia was identified as significant risk factors for colorectal polyps in patients with NAFLD (OR 2.04). Dyslipidemia may promote the development and progression of colorectal polyps and tumors through multiple mechanisms, including activation of insulin-like growth factor 1 (IGF-1) expression, induction of inflammatory cytokines (such as IL-6 and TNF-α), stimulation of bile acid secretion, oxidative stress, and DNA damage. NAFLD includes a spectrum of conditions, from simple fatty liver to steatohepatitis, fibrosis, and cirrhosis. The incidence of colorectal polyps varies across these different stages of disease progression. Mahmud Mahamid MD found that presence of NASH was associated with increased risk for colorectal polyps (OR 1.69, 95%CI 1.36–1.98, *P* < 0.01) (18). This may be attributed to the more severe inflammatory response, insulin resistance, and gut microbiota dysbiosis associated with NASH.

This study constructed a moderate predictive model for colorectal polyp risk in patients with NAFLD based on findings from a meta-analysis and logistic regression analysis. By quantifying risk factors identified from an initial pool of 15 studies, the model facilitates rapid patient risk assessment. Through comprehensive analysis, 6 distinct risk factors associated with polyp development in NAFLD patients were ultimately identified. Additionally, we validated the colorectal polyp risk prediction model using an internal cohort of 609 patients with NAFLD selected from hospital. The model demonstrated moderate discriminatory ability, with an AUC of 0.73 and 0.71.

To our knowledge, this study represents the first attempt to construct a predictive model for colorectal polyps in NAFLD patients based on risk factors identified via meta-analysis, LASSO regression and multivariable logistic regression. The model offers a practical tool for clinicians to identify high-risk patients and inform prevention strategies for colorectal tumors. Nevertheless, the study has limitations. Firstly, Selection of the study population. In the meta-analysis, we obtained the candidate predictive factors from the literature for different countries and regions. With 11 of the 15 studies conducted in Asian populations, the model requires external validation in non-Asian cohorts to confirm its generalizability. In retrospective cohort, we mainly selected patients who had undergone colonoscopy during their hospital stay and who suffered from NAFLD. The inpatients in our department are mostly elderly individuals, or patients who already have intestinal symptoms such as abdominal pain, diarrhea, abdominal distension, changes in bowel habits, etc. These factors all led to a high detection rate of colon polyps. Therefore, expanding the scope of the included population, such as including the healthy examination population from outpatient departments and health checkup centers, can reduce the bias in the included population. Additionally, excluding factors reported in fewer than three studies may have led to the omission of potentially relevant predictors. Therefore, it is necessary to update the included literature in a timely manner. Finally, this model was developed and validated based on a single-center sample, which highlights the necessity of conducting external validation in a prospective, multi-center, large-sample group setting.

## 5 Conclusion

This study employed meta-analysis, LASSO regression and multivariable logistic regression analysis to identify 6 significant risk factors for colorectal polyps in the NAFLD population: age ( ≥ 50), male sex, obesity, diabetes, hypertriglyceridemia, and fatty liver type. A predictive model incorporating these factors was developed and subsequently validated using internal validation. The resulting tool enables effective risk prediction, supporting early clinical identification and stratified management of NAFLD patients at high risk for colorectal polyps.

## Data Availability

The raw data supporting the conclusions of this article will be made available by the authors, without undue reservation.
